# Crystallographic and thermodynamic characterization of phenylaminopyridine bisphosphonates binding to human farnesyl pyrophosphate synthase

**DOI:** 10.1371/journal.pone.0186447

**Published:** 2017-10-16

**Authors:** Jaeok Park, Dmitry Rodionov, Joris W. De Schutter, Yih-Shyan Lin, Youla S. Tsantrizos, Albert M. Berghuis

**Affiliations:** 1 Department of Biochemistry, McGill University, Montreal, Quebec, Canada; 2 Department of Chemistry, McGill University, Montreal, Quebec, Canada; University of Queensland, AUSTRALIA

## Abstract

Human farnesyl pyrophosphate synthase (hFPPS) catalyzes the production of the 15-carbon isoprenoid farnesyl pyrophosphate. The enzyme is a key regulator of the mevalonate pathway and a well-established drug target. Notably, it was elucidated as the molecular target of nitrogen-containing bisphosphonates, a class of drugs that have been widely successful against bone resorption disorders. More recently, research has focused on the anticancer effects of these inhibitors. In order to achieve increased non-skeletal tissue exposure, we created phenylaminopyridine bisphosphonates (P*N*P-BPs) that have bulky hydrophobic side chains through a structure-based approach. Some of these compounds have proven to be more potent than the current clinical drugs in a number of antiproliferation assays using multiple myeloma cell lines. In the present work, we characterized the binding of our most potent P*N*P-BPs to the target enzyme, hFPPS. Co-crystal structures demonstrate that the molecular interactions designed to elicit tighter binding are indeed established. We carried out thermodynamic studies as well; the newly introduced protein-ligand interactions are clearly reflected in the enthalpy of binding measured, which is more favorable for the new P*N*P-BPs than for the lead compound. These studies also indicate that the affinity of the P*N*P-BPs to hFPPS is comparable to that of the current drug risedronate. Risedronate forms additional polar interactions via its hydroxyl functional group and thus exhibits more favorable binding enthalpy; however, the entropy of binding is more favorable for the P*N*P-BPs, owing to the greater desolvation effects resulting from their large hydrophobic side chains. These results therefore confirm the overall validity of our drug design strategy. With a distinctly different molecular scaffold, the P*N*P-BPs described in this report represent an interesting new group of future drug candidates. Further investigation should follow to characterize the tissue distribution profile and assess the potential clinical benefits of these compounds.

## Introduction

**Fig 1:**
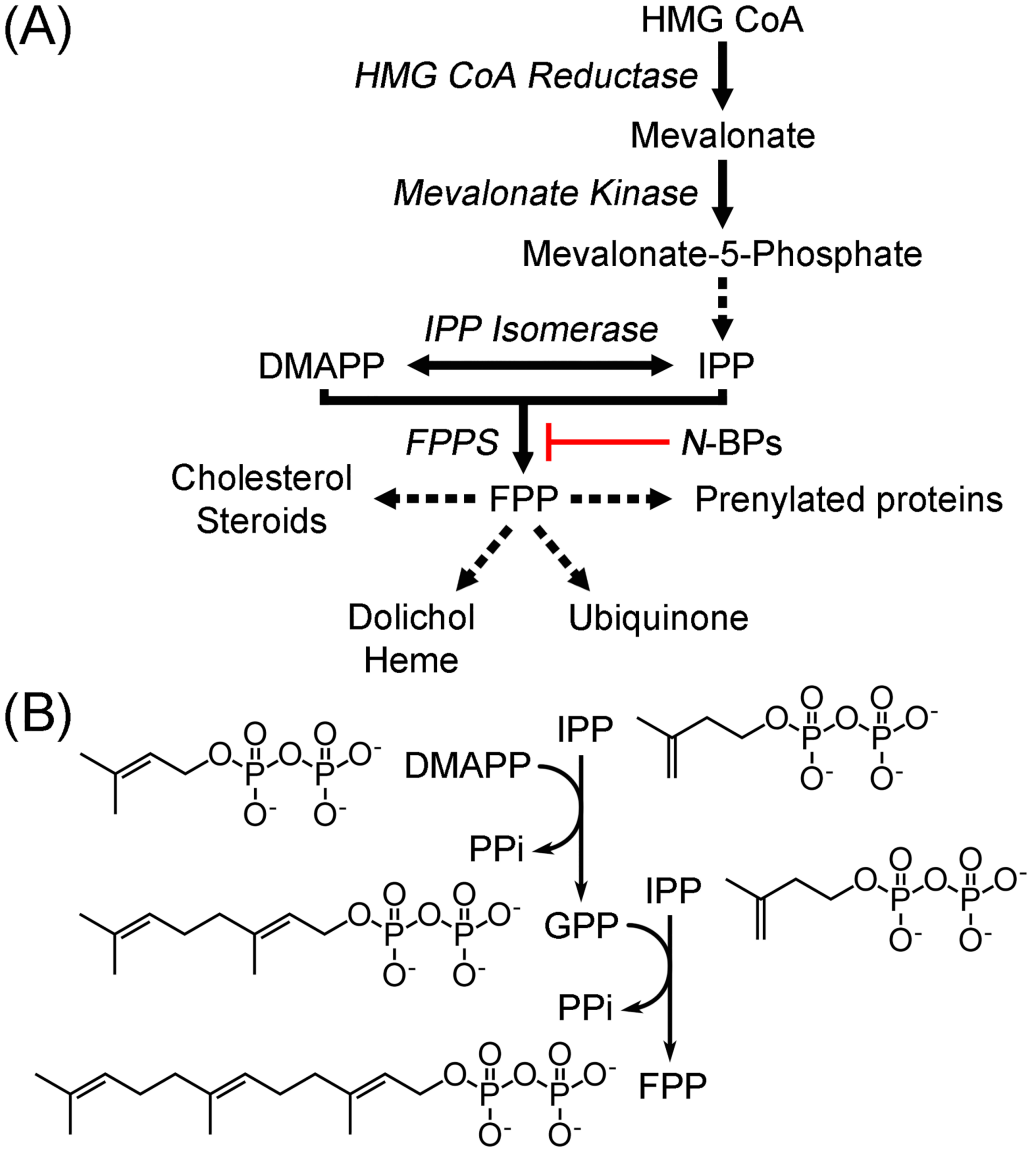
Mevalonate pathway and FPP synthesis. (A) Overview of mevalonate pathway and downstream metabolites. Enzyme names are in Italics. Dotted arrows represent multi-enzyme processes. (B) Reactions carried out by hFPPS.

Human farnesyl pyrophosphate synthase (hFPPS) is a homodimeric enzyme with a subunit molecular mass of 42 kDa. Occupying the first branching point in the mevalonate pathway (Fig 1A), the enzyme catalyzes the sequential chain elongation of dimethylallyl pyrophosphate (DMAPP) to geranyl pyrophosphate (GPP) and then to farnesyl pyrophosphate (FPP) through successive condensation with two molecules of isopentenyl pyrophosphate (IPP) (Fig 1B). FPP is absolutely required for prenylation of small GTPases, which is essential for the subcellular localization and function of these proteins [1]. Osteoclast death via inhibition of hFPPS function and consequently that of small GTPase prenylation has been well established as the mechanism of action for the potent antiresorptive activity of nitrogen-containing bisphosphonate (N-BP) drugs (Fig 1A) [2].

Small GTPases include proto-oncogenic Ras family members implicated in a plethora of cancers. Their transforming activity still requires prenylation, and thus inhibiting this process is thought to be a promising therapeutic strategy [3]. Indeed, a large body of preclinical and clinical evidence indicates that N-BPs also have anticancer activity; they inhibit proliferation, motility, and viability of tumor cells, and act in synergy with other antineoplastic agents [4]. In addition to prenylation blockade, other, indirect mechanisms may contribute to the observed anticancer effects. Inhibition of hFPPS results in cellular accumulation of IPP, which induces apoptosis in cancer cells via its cytotoxic metabolite (i.e., ApppI, the isopentenyl ester of ATP) as well as activates T immune cells that can seek and destroy cancer cells [5].

**Fig 2:**
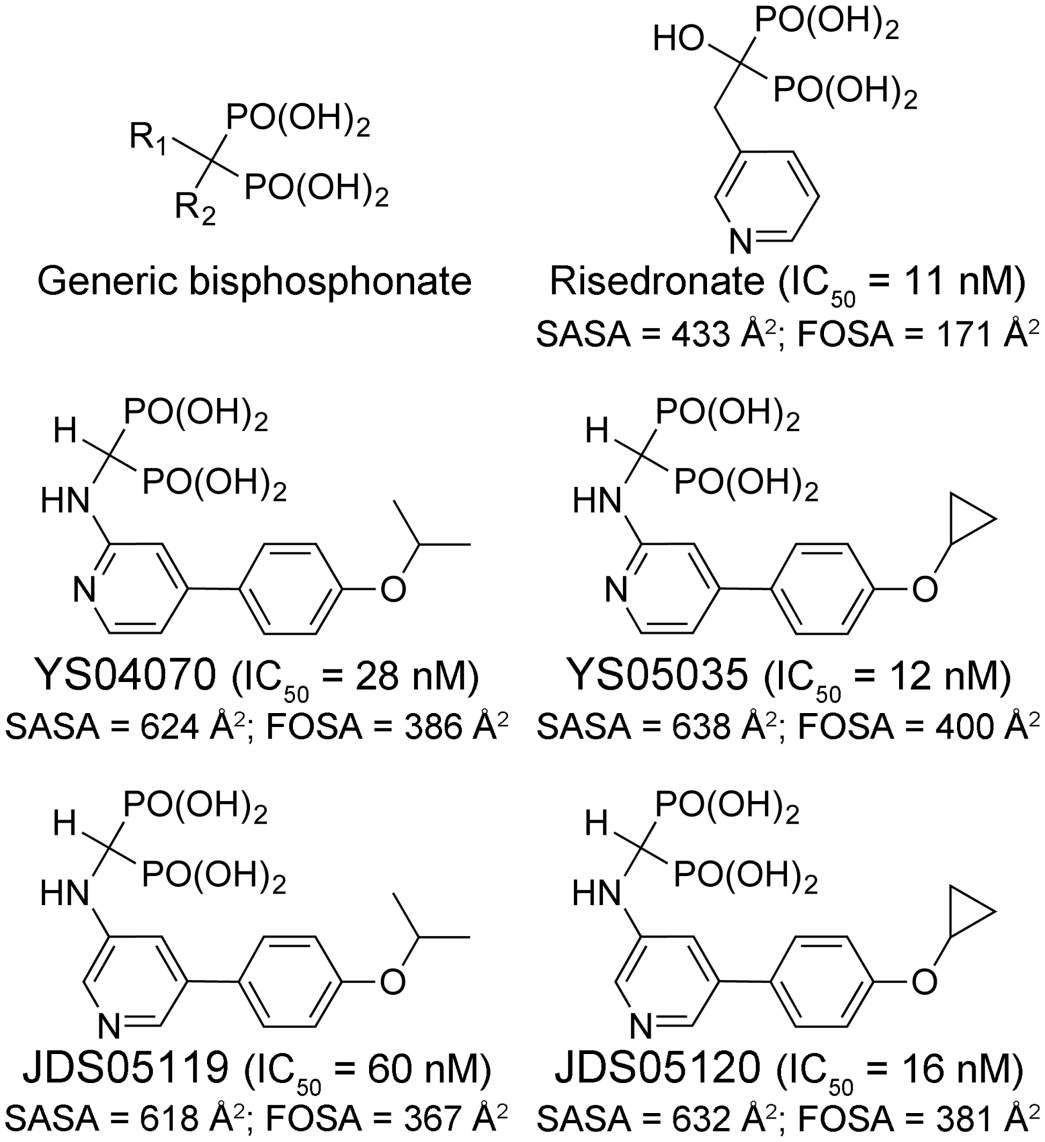
Bisphosphonate inhibitors of hFPPS. Carbon hydrogens in the R_2_ side chains are omitted for simplicity. IC_50_ values were reported previously [9, 10]. SASA (total solvent accessible surface area) and FOSA (hydrophobic component of SASA) were calculated with QikProp 3.2 by using a virtual probe of 1.4 Å radius.

**Fig 3:**
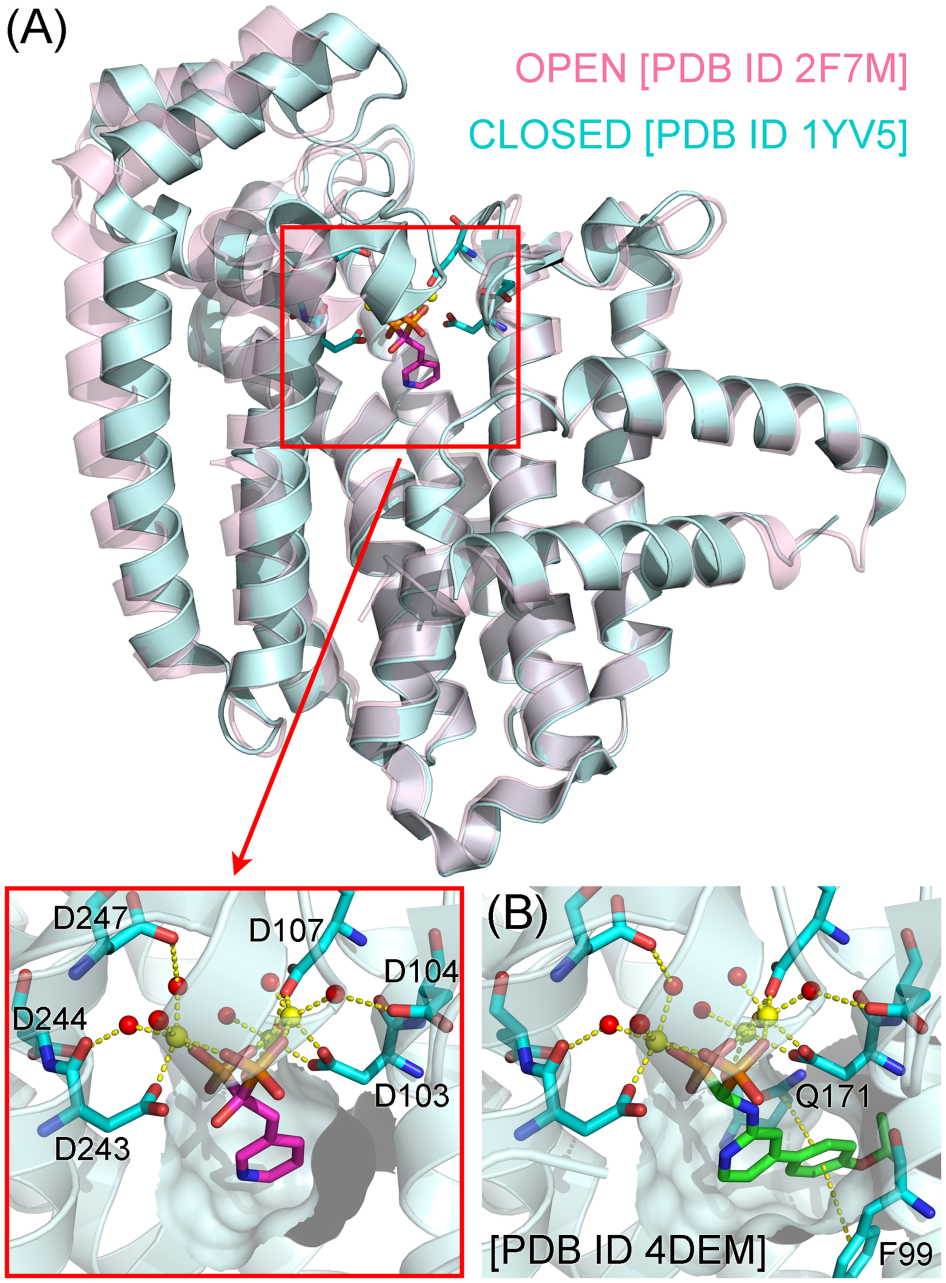
Binding of bisphosphonates risedronate (A) and YS04070 (B) to hFPPS. (A) The enzyme undergoes an open-to-closed conformational change upon DMAPP/GPP or bisphosphonate binding. This enzyme closure fully shapes the second substrate binding site, the IPP subpocket. The inset shows details of the Mg^2+^ (yellow spheres)-mediated binding interactions between the bisphosphonate moiety of risedronate and the DDXXD motifs. Mg^2+^-coordinated water molecules (red spheres) and the active site hydrophobic cavity (surface) are also represented. Only one subunit of the homodimer is shown for clarity. (B) The hydrophobic cavity accommodates the bulky side chain of YS04070. The Mg^2+^-mediated interactions are identical to those seen with risedronate.

The physicochemical properties of the current N-BP drugs (e.g., risedronate, Fig 2), however, compromise their antineoplastic potential in non-skeletal tissues. They have poor membrane permeability and oral bioavailability, and once in the systemic circulation, bind rapidly and almost exclusively to bone [6]. This is due mainly to their negatively charged bisphosphonate moiety, which mimics the pyrophosphate of hFPPS substrates. N-BPs bind to the DMAPP/GPP subpocket of the hFPPS active site, with their bisphosphonate group coordinated to the Asp-rich (DDXXD) motifs of the enzyme via three Mg^2+^ ions (Fig 3A) [7, 8]. The DMAPP/GPP subpocket also consists of a sizable hydrophobic cavity, which accommodates the prenyl tail of the substrates or the R_2_ side chain of N-BPs (Fig 3A; see Fig 2 for the general structure of bisphosphonates).

In an effort to identify hFPPS inhibitors that can better target non-skeletal tissues, we designed and synthesized a library of aminopyridine-based bisphosphonates with bulkier and more lipophilic side chains than those of the current N-BP drugs. Some of the lead compounds showed greater potency than risedronate in a number of multiple myeloma cell lines despite higher IC_50_ values, with the improvement stemming from increased hydrophobicity and membrane permeability [9]. A co-crystal structure (Protein Data Bank (PDB) entry 4DEM) with a representative inhibitor, YS04070 (Fig 2), showed that its R_2_ side chain fully occupies the hFPPS active site hydrophobic cavity, participating in unique π-stacking interactions with the residues Phe99 and Gln171 (Fig 3B) [9]. Through an *in silico* docking approach exploiting this structure, we synthesized additional phenylaminopyridine bisphosphonates (P*N*P-BPs) designed to engage in targeted interactions with other active site residues [10]. In order to examine the actual binding interactions between the new P*N*P-BPs and hFPPS, we now determine co-crystal structures with the key inhibitors YS05035, JDS05119, and JDS05120 (Fig 2). Complimentary solution binding profiles are also determined by isothermal titration calorimetry (ITC).

## Materials and methods

### Human FPPS and P*N*P-BPs

The expression and purification of the protein, as well as the synthesis of the bisphosphonates, have been described in detail previously [9, 10].

### Crystallization

P*N*P-BPs were prepared as 20 mM solutions in 100 mM Tris-HCl pH 7.5, and MgCl_2_ as a 100 mM aqueous solution. The bisphosphonate and MgCl_2_ solution were added to the purified hFPPS sample in various concentrations (see Table 1 for details). The final concentration of the protein was 0.25 mM in all cases. Crystals were obtained at 295 K by vapor diffusion in sitting drops composed of 1–1.5 μL protein sample and 1 μL crystallization buffer, and additional 0.5 μL microseed solution when added (Table 1). Seed stocks were prepared with Seed Bead kits (Hampton Research) and crystals that were deemed too small for data collection. Typically about five crystals were added to a seed bead tube containing 50 μL reservoir solution that produced the crystals, which were then crushed by vortexing the tube for 3 minutes. The crushed crystals were added with 450 μL fresh solution having the same composition as the reservoir solution and mixed thoroughly by vortexing for 3 minutes again. The 500 μL stock solutions were stored at room temperature and diluted 10^2^−10^4^ times when used in subsequent crystallization trials.

**Table 1:** Crystallization conditions.

Data set^a^	YS05035	JDS05119	JDS05120-1	JDS05120-2	JDS05120-3
Composition of protein solution	0.01 M HEPES (pH 7.5), 0.5 M NaCl, 0.02 M βME, 5% glycerol, 3 mM MgCl_2_, 1 mM YS05035	0.01 M HEPES (pH 7.5), 0.5 M NaCl, 0.02 M βME, 5% glycerol, 3 mM MgCl_2_, 1 mM JDS05119	0.01 M HEPES (pH 7.5), 0.5 M NaCl, 0.02 M βME, 5% glycerol, 1.5 mM MgCl_2_, 3 mM JDS05120	0.01 M HEPES (pH 7.5), 0.5 M NaCl, 0.02 M βME, 5% glycerol, 1.5 mM MgCl_2_, 1 mM JDS05120	0.01 M HEPES (pH 7.5), 0.5 M NaCl, 0.02 M βME, 5% glycerol, 3 mM JDS05120
Composition of reservoir solution	5.6% PEG 4K, 30% glycerol, 0.07 M NaCH_3_COO (pH 4.6)	1.7 M NaCl, 15% glycerol, 0.085 M NaCH_3_COO (pH 4.6)	20% PEG 3.35K, 0.2 M Mg(HCO_2_)_2_ (pH 5.9)	0.17 M (NH_4_)_2_SO_4_, 25.5% PEG MME 2K, 15% glycerol, 0.09 M NaCH_3_COO (pH 4.6)	0.01 M NiCl_2_, 0.85 M Li_2_SO_4_, 15% glycerol, 0.09 M Tris (pH 8.5)
Composition of reservoir solution used to obtain microseeds	N/A	N/A	N/A	0.16 M Mg(CH_3_COO)_2_, 16% PEG 8K, 20% glycerol, 0.08 M Na(CH_3_)_2_AsO_2_ (pH 6.5)	0.8 M KH_2_PO_4_, 0.8 M NaH_2_PO_4_, 0.1 M HEPES (pH 7.5)
Volume of reservoir, protein, and seed solution in crystallization drop (μL)	1:1.5	1:1	1:1	1:1:0.5	1:1:0.5
Volume of reservoir (μL)	80	80	80	80	80

^a^Five single crystals were used for subsequent data collection, three of which contained JDS05120 but were obtained under different conditions.

### Data collection and processing and structure refinement

Diffraction data were collected from single crystals at 100 K either with a synchrotron radiation source and a Rayonix MX300 CCD detector (Beamline 08ID-1, Canadian Light Source, Saskatoon, SK, Canada) or with a MicroMax-007 HF generator (Rigaku) and a Saturn 994+ CCD detector (Rigaku). All data sets were processed with the *xia*2 program package [11]. The structure models were initially built by a difference Fourier method with a ligand/solvent-omitted starting model generated from the PDB entry 4H5C [12]. The models were improved through iterative rounds of manual and automated refinement with the programs *Coot* [13] and *REFMAC*5 [14]. Stereochemical restraints for the bisphosphonate ligands were obtained from the *PRODRG*2 server [15]. The final models were deposited in the PDB (the validation reports are included as S1–S5 Appendices). Data collection and structure refinement statistics are summarized in Table 2.

**Table 2:** Data collection and structure refinement statistics.

Data set	YS05035	JDS05119	JDS05120-1	JDS05120-2	JDS05120-3
PDB code	4PVX	4PVY	4NFI	4NFJ	4NFK
**Data collection**					
Oscillation range (° frames^-1^)	0.5	0.25	0.40	0.50	0.30
No. of frames	1884	3780	299	239	400
Space group	*P*4_1_2_1_2	*P*4_1_2_1_2	*P*4_1_2_1_2	*P*4_1_2_1_2	*P*4_1_2_1_2
Unit cell dimension (Å)	*a* = *b* = 111.26, *c* = 69.34	*a* = *b* = 111.06, *c* = 67.03	*a* = *b* = 110.51, *c* = 66.97	*a* = *b* = 111.60, *c* = 68.10	*a* = *b* = 110.94, *c* = 68.46
Resolution range (Å)	69.34–2.18 (2.24–2.18)	111.06–2.05 (2.10–2.05)	50.85–1.85 (1.90–1.85)	51.56–2.05 (2.11–2.05)	51.58–1.85 (1.90–1.85)
Completeness (%)	98.7 (98.9)	98.8 (90.9)	99.4 (99.4)	99.5 (98.0)	99.0 (97.6)
Redundancy	37.9 (10.9)	32.9 (3.0)	9.6 (9.7)	9.7 (9.7)	9.8 (9.6)
*I*/σ(*I*)	53.8 (3.5)	44.1 (2.5)	29.5 (5.0)	28.9 (4.5)	25.8 (4.7)
*R*_merge_	0.062 (0.653)	0.067 (0.397)	0.042 (0.437)	0.042 (0.461)	0.049 (0.470)
CC_1/2_	1.000 (0.909)	1.000 (0.840)	1.000 (0.949)	0.999 (0.940)	0.999 (0.935)
**Refinement**					
No. of reflections	21774	25246	33802	25777	34008
*R*_work_/*R*_free_	0.170/0.220	0.160/0.206	0.165/0.196	0.174/0.213	0.152/0.182
No. of non-H atoms					
Protein	2751	2769	2748	2709	2745
Ion	3	3	3	3	3
Ligand	32	32	26	31	31
Water	148	241	205	105	220
Total	2934	3045	2982	2848	2999
Average *B* factor (Å^2^)					
Protein	39.31	32.54	37.51	47.16	39.27
Ion	27.02	20.67	23.09	45.22	26.93
Ligand	35.67	25.47	28.32	47.40	31.57
Water	39.10	37.74	41.57	46.16	43.33
R.m.s. deviations					
Bonds (Å)	0.017	0.019	0.019	0.018	0.019
Angles (°)	1.7	1.8	1.9	1.9	1.8
Ramachandran plot^a^					
Most favoured (%)	98.8	99.1	99.1	98.8	99.4
Allowed (%)	1.2	0.9	0.9	1.2	0.6

Values for the highest resolution shell are given in parentheses.

### Isothermal titration calorimetry

ITC experiments were carried out at 303 K with a MicroCal iTC_200_ system (GE Healthcare Life Sciences). The hFPPS and P*N*P-BP solutions were prepared in the same buffer (10 mM HEPES pH 7.5, 500 mM NaCl, 2 mM βME, and 5% glycerol) at 0.1 and 1 mM concentrations, respectively. The protein sample was saturated with 10 mM MgCl_2_ prior to titration. Each titration experiment consisted of one 1 μL injection followed by eighteen 2 μL injections of a bisphosphonate solution into 200 μL protein sample. Heats of dilution were measured in control experiments and subtracted from the actual titration data. With every P*N*P-BP, three independent titration experiments were carried out. The mean data were analyzed by fitting to the one-site binding model implemented in the Origin 7 software (OriginLab) [16, 17].

## Results and discussion

**Fig 4:**
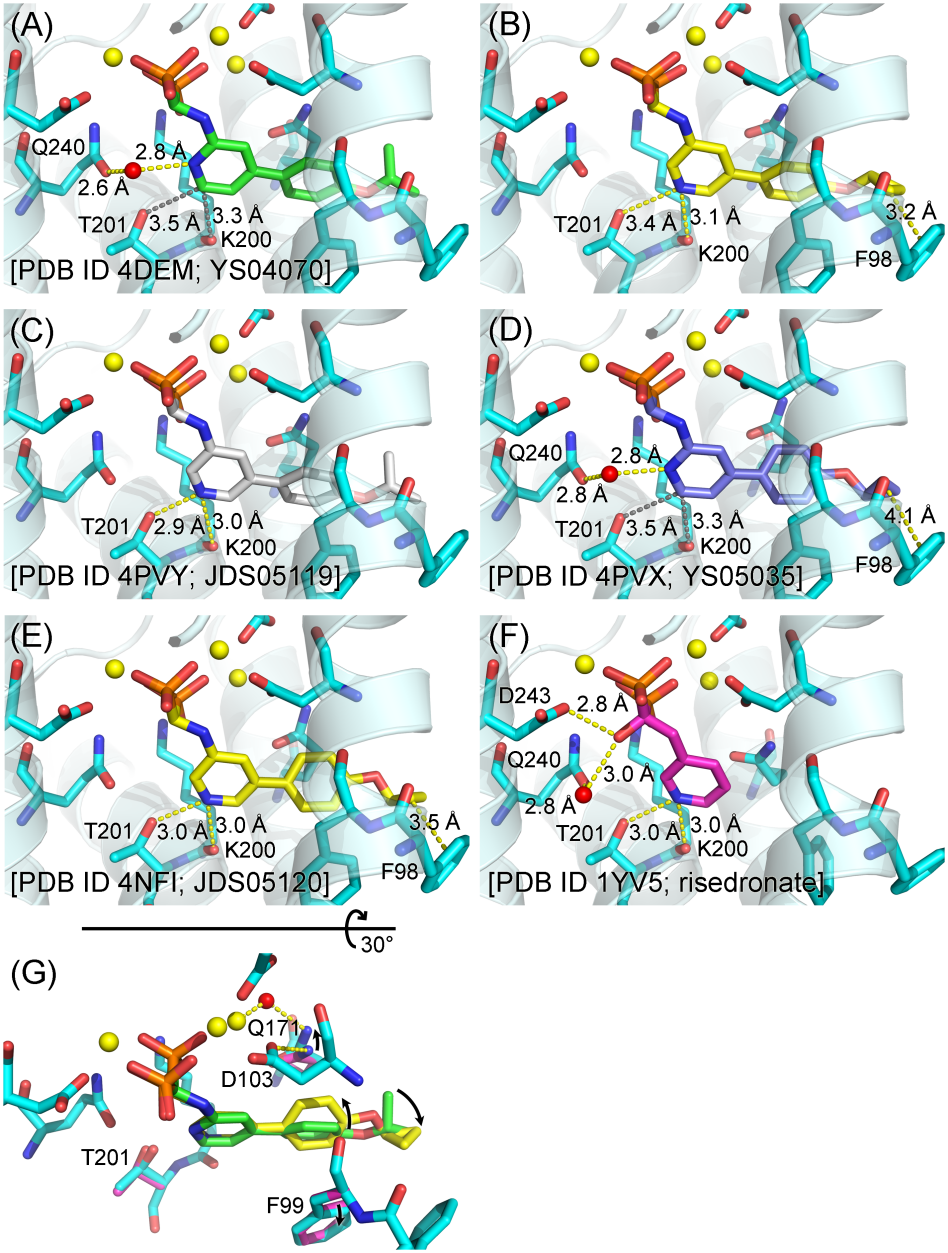
Binding of P*N*P-BPs and risedronate to hFPPS. (A) Co-crystal structure with YS04070. (B) Docking output structure with JDS05120 [10]. (C), (D), and (E) Co-crystal structures with the new inhibitors JDS05119, YS05035, and JDS05120, respectively. See S1 Fig for the ligand discovery maps. The two additional structures with JDS05120 (PDB entries 4NFJ and 4NFK, Table 2) do not show significant differences and are thus not shown. (F) Co-crystal structure with risedronate. (G) The JDS05120-bound structure is superimposed onto the YS04070-bound structure (protein residues in magenta; YS04070 in green). The conformational differences in the key residues and inhibitors are indicated. The cartoon representation of the protein is omitted for unobstructed view of the bound bisphosphonates. Select H-bond interactions are shown as yellow dashed lines, while relevant non-H-bond distances are indicated by grey dashed lines.

We explored two structural modifications of the lead inhibitor YS04070 with the new P*N*P-BPs YS05035, JDS05119, and JDS05120. In the hFPPS/YS04070 complex, the pyridyl nitrogen of the bisphosphonate participates in a water-mediated H-bond with the side chain oxygen of Gln240 (Fig 4A). The position of the pyridyl nitrogen was changed in JDS05119 and JDS05120 (thus making them 3-aminopyridine bisphosphonates), so that the nitrogen could form a bifurcated H-bond upon protonation with the main chain oxygen of Lys200 and the side chain oxygen of Thr201 (Fig 4B). An analogous H-bond is thought to contribute significantly to the potency of risedronate [18]. In addition, the isopropyl tail of the YS04070 side chain was replaced with a cyclopropyl group in YS05035 and JDS05120. Due to the strain in the three-membered ring system, the C-C bonds in the cyclopropyl group are “bent” and do not have a normal sigma bond [19]. Despite still being single bonds, they possess π character (i.e., the density of the bonding electrons lies off the internuclear axis) and could thus provide a favorable stacking interaction with the aromatic ring of Phe98. Our docking study predicted the new substituent to position within van der Waals distance from the side chain of Phe98 (closest carbon to carbon distance of 3.2 Å, Fig 4B) [10].

### Crystal structures of hFPPS/P*N*P-BP complexes

The present crystal structures clearly demonstrate the new binding interaction introduced by the pyridyl nitrogen in JDS05119 and JDS05120: the side chain of Thr201 is attracted towards the pyridine ring, and the bifurcated H-bond distances are ~3 Å (Fig 4C and 4E), within the optimal range. These distances are consistent in the crystals obtained under different conditions covering the ~5–8 pH range (see Table 2 for crystallization details) and thus expectedly of physiological relevance. In contrast, the equivalent distances with the lead compound YS04070 are 3.5 and 3.3 Å (Fig 4A). These distances remain the same with the new 2-aminopyridine analog YS05035, which also retains the water-mediated H-bond to Gln240 (Fig 4D). It should be noted that risedronate makes a similar water-mediated interaction to Gln240 despite the position of its pyridyl nitrogen, via the R_1_ hydroxyl substituent (Fig 4F). In addition, this hydroxyl group forms a direct H-bond with Asp243 (Fig 4F). Simultaneous polar interactions with Lys200, Thr201, and Gln240 mimic those that stabilize the substrate carbocation intermediate during catalysis [20] and likely contribute to the high potency of risedronate. Our series of P*N*P-BPs lacks the R_1_ hydroxyl moiety by design, since this functional group significantly increases the binding affinity of bisphosphonates for bone mineral [21] (and is thus commonly referred to as the “bone hook”).

Unlike the optimization effort with the pyridyl nitrogen, it is unclear from the current structures whether introducing the cyclopropyl tail results in the expected binding interaction. The predicted proximity between the bisphosphonate tail and Phe98 is confirmed with JDS05120 (3.5 Å, Fig 4E); however, with YS05035 the distance is 4.1 Å (Fig 4D), slightly over the generally accepted π-interaction limit of 4 Å. The only difference between the two compounds is the location of the pyridyl nitrogen. This observation suggests that the bifurcated H-bond helps position the cyclopropyl tail closer to Phe98 and is thus required for the intended CH/π-interaction. A 0.3 Å movement of the pyridyl core translating into a 0.6 Å movement of the propyl tail is not surprising. The co-crystal structures also reveal conformational changes in both the enzyme and inhibitors that could not be predicted by the docking experiments. In contrast to those of their isopropyl analogs (and those in their docked poses), the tails of YS05035 and JDS05120 are flipped down at the ether linkage (see Fig 4G for this difference). In addition, the preceding phenyl moieties are rotated with respect to the pyridine plane to the opposite direction (Fig 4G). The dihedral angles between the pyridine and phenyl rings in the cyclopropyl P*N*P-BPs are larger than those in their isopropyl analogs (~35 vs. <25°) and closer to the 40–45° range calculated to yield the lowest torsional potential for similar phenylpyridine systems [22]. In the protein, the side chains of Phe99 and Gln171 assume slightly different conformations to accommodate the new phenylpyridine configuration; in particular, the side chain nitrogen of Gln171 now forms a H-bond with one of the Mg^2+^-coordinated water molecules instead of the side chain oxygen of Asp103 (Fig 4G).

### Thermodynamic characterization of hFPPS and P*N*P-BP binding

**Fig 5:**
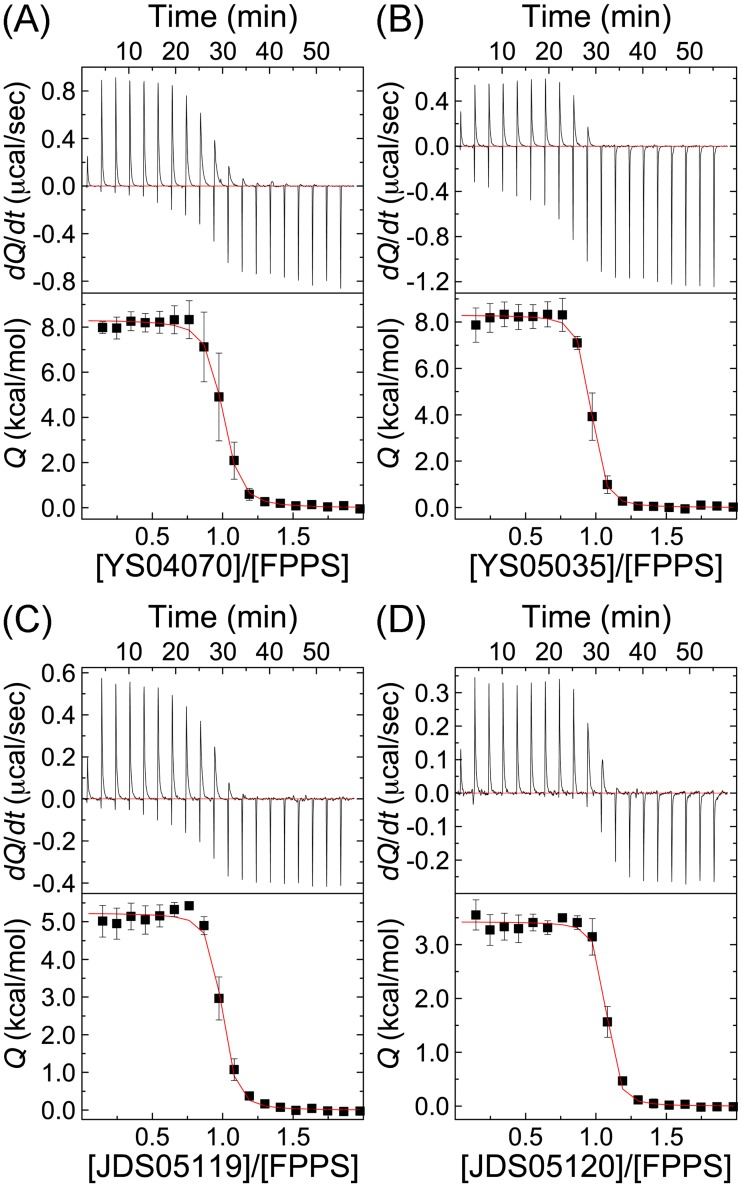
ITC characterization of hFPPS and P*N*P-BP binding. (A) YS04070, (B) YS05035, (C) JDS05119, and (D) JDS05120. The upper panels present raw thermograms; for clarity, only a single representative run is shown for each compound. The lower panels present the binding isotherms fitted to the means of three independent experiments. SE values are shown as bars.

The P*N*P-BPs bind to hFPPS in an endothermic process (Fig 5), as also observed for risedronate previously [8]. The binding is entropically driven, with a positive, unfavorable enthalpy change (*T*Δ*S* >Δ*H*, Table 3). This is in sharp contrast to that seen with the substrates DMAPP and GPP, the binding of which at the same site is exothermic and enthalpically driven [23]. The differences in the enthalpy of binding between the individual bisphosphonates well explain the protein-ligand interactions confirmed crystallographically. The lower, more favorable Δ*H* with JDS05119 and JDS05120 (compared to their 2-aminopyridine counterpart, YS04070 and YS05035, respectively; Table 3) likely reflects the bifurcated H-bond via the pyridyl nitrogen. Between the 3-aminopyridine P*N*P-BPs, JDS05120 shows more favorable Δ*H* (Table 3), consistent with the CH/π-interaction introduced by the cyclopropyl substitution. On the other hand, the enthalpy of binding does not differ significantly between the 2-aminopyridine analogs (Table 3). This is because YS05035, despite having a cyclopropyl tail, cannot effectively form an analogous CH/π-interaction; as discussed earlier, the distance from its tail to Phe98 is above the upper limit to allow such an interaction. Risedronate shows the most favorable binding enthalpy (Table 3), which probably owes to the additional polar interactions via the R_1_ hydroxyl moiety.

**Table 3:** Thermodynamic parameters of hFPPS and bisphosphonate binding determined by ITC.

Ligand	*N*	*K*_d_ (nM)	Δ*H* (kcal/mol)	*T*Δ*S* (kcal/mol)
YS04070	0.95 ± 0.01	325 ± 57	8.31 ± 0.08	17.31
YS05035	0.92 ± 0.004	172 ± 33	8.30 ± 0.07	17.67
JDS05119	0.95 ± 0.01	197 ± 58	5.23 ± 0.07	14.52
JDS05120	1.03 ± 0.01	120 ± 36	3.43 ± 0.04	13.07
Risedronate [8]^a^	1.14	164 ± 54	1.8 ± 0.4	10.5

The parameters *N* (binding stoichiometry), *K*_d_, and Δ*H* were determined by least squares curve fitting; deviations represent standard errors derived from the curve fitting.

The entropic contribution (*T*Δ*S*) to the binding free energy (Δ*G*) was calculated based on the following relationships: Δ*G* = − *RT* ln 1/*K*_d_ = Δ*H* − *T*Δ*S*, where *R* is the universal gas constant, and *T* is the temperature in Kelvin.

Values are per monomer of the enzyme.

^a^Described previously.

The newly introduced protein-ligand interactions, however, do not directly translate into the binding affinity of the P*N*P-BPs. For example, despite the large enthalpic gain from the bifurcated H-bond (>3 kcal/mol), the *K*_d_ values of the 3-aminopyridine P*N*P-BPs are only ~1.5-fold lower than those of their respective 2-aminopyridine analogs (compare JDS05119 to YS04070 and JDS05120 to YS05035, Table 3). The modest improvement in affinity is due to the losses in the entropic component of binding (*T*Δ*S*, Table 3), which offset the enthalpic gains from the energetically favorable protein-ligand interactions. This optimization effort thus exemplifies the paradoxical phenomenon of entropy-enthalpy compensation: the entropic and enthalpic contributions change substantially as the lead is modified but in an antagonistic manner that changes the overall binding free energy only slightly. The compensation effects apply to risedronate as well. Despite having the most favorable Δ*H*, risedronate has its *K*_d_ value in the same range as the new P*N*P-BPs, with *T*Δ*S* that is the least favorable by a similar margin (Table 3). The entropy of binding can be parsed into three components. The loss of rotational/translational freedom of the protein and ligand contributes unfavorably and should not differ significantly based on the binding bisphosphonate. Changes in the conformational freedom should also be unfavorable (hFPPS and the bisphosphonates adopt more rigid conformations upon complex formation) and similar across (especially for the binding of the P*N*P-BPs). The last component is the largely favorable gain in solvent entropy, which arises from the release of water molecules from the surface of the enzyme and bisphosphonates upon their binding (i.e., desolvation). In particular, desolvation of the ligand would be the major factor that determines the differences in the binding entropy here. How favorable the entropy of binding is for the P*N*P-BPs and risedronate coincides well with the size of their solvent accessible hydrophobic surface area (FOSA, Fig 2).

Overall, the binding affinity of the bisphosphonates corresponds well with their inhibitory activity; risedronate and the cyclopropyl P*N*P-BPs show similar potency, and the isopropyl analogs slightly lower. However, the difference between the *K*_d_ and IC_50_ values for each compound is quite significant (IC_50_’s are on average ~10-fold lower than the *K*_d_’s; Fig 2 and Table 3). The most likely explanation for the discrepancy is that during inhibition assays the second substrate IPP binds to the hFPPS-bisphosphonate complex and traps the inhibitor inside the enzyme by forming a stable ternary complex (i.e., the hFPPS-bisphosphonate-IPP complex; the transient, catalytically active equivalent is the hFPPS-DMAPP-IPP or hFPPS-GPP-IPP complex). Here the competition between the bound inhibitor and free DMAPP/GPP is inefficient, and this amplifies the potency of the bisphosphonate compounds. The binding of IPP to the hFPPS-bisphosphonate complex and the subsequent increase in the thermal stability of the complex have been observed previously for several N-BPs including risedronate and the P*N*P-BP YS04070 [7, 12].

## Conclusions

We examined the binding of our most potent P*N*P-BP inhibitors to hFPPS in this work. The presence of the protein-ligand interactions designed to achieve tighter binding have been confirmed by the crystallographic data. The details of the ligand binding, however, slightly differ from those predicted in the previous docking study [10]. These results demonstrate the usefulness of *in silico* docking in lead optimization but also its limitations in dealing with protein/ligand flexibility. The ITC data explains the structural observations very well. The enthalpy of binding is more favorable for the 3-aminopyridine P*N*P-BPs than for the 2-aminopyridine analogs, reflecting the new protein-ligand interactions introduced; it is less favorable than for risedronate, consistent with the lack of the hydroxyl bone hook. Nevertheless, the binding affinity of the new P*N*P-BPs is similar to that of risedronate. This is because the entropic component of the overall binding energy is more favorable for the P*N*P-BPs and able to compensate the enthalpic deficit. Having comparable inhibitory potency towards hFPPS but also different physicochemical properties compared to the current drugs, the P*N*P-BPs reported here make interesting candidates worth studying for their non-skeletal clinical benefits. Further optimization and biological evaluation of these inhibitors are thus warranted.

## Supporting information

S1 Appendix PDB validation report for the entry 4PVX. (PDF)

S2 Appendix PDB validation report for the entry 4PVY. (PDF)

S3 Appendix PDB validation report for the entry 4NFI. (PDF)

S4 Appendix PDB validation report for the entry 4NFJ. (PDF)

S5 Appendix PDB validation report for the entry 4NFK. (PDF)

S1 Fig Discovery maps for P*N*P-BPs. (A) JDS05119 (PDB entry 4PVY); (B) YS05035 (PDB entry 4PVX); (C), (D), and (E) JDS05120 (PDB entries 4NFI, 4NFJ, and 4NFK, respectively). The green meshes represent the *F*_o_-*F*_c_ electron density maps (3σ) generated by Fourier synthesis before ever modeling the ligands. Green spheres are Ni^2+^ ions.
(PDF)
