# Supplementary material for: Crystallographic and thermodynamic characterization of phenylaminopyridine bisphosphonates binding to human farnesyl pyrophosphate synthase
Source: PLoS One. 2017 Oct 16;12(10):e0186447. doi: 10.1371/journal.pone.0186447 (PMC5643135; doi:10.1371/journal.pone.0186447)
Supplement: S3 Appendix — (PDF) [file pone.0186447.s003.pdf]

wwPDB X-ray Structure Validation Report (i)

Jan 15, 2014 – 12:39 PM EST

PDB ID	:	4NFI
Title	:	Crystal structure of human FPPS in complex with magnesium and JDS05120
Authors	:	Park, J.; De Schutter, J.W.; Tsantrizos, Y.S.; Berghuis, A.M.
Deposited on	:	2013-10-31
Resolution	:	1.85 Å(reported)

DISCLAIMER
This is a preliminary version of the new style of wwPDB validation report.
We welcome your comments at validation@mail.wwpdb.org
A user guide is available at http://wwpdb.org/ValidationPDFNotes.html

CONFIDENTIAL VALIDATION REPORT

The following versions of software and data (see references) were used in the production of this report:

MolProbity	:	4.02b-467
Mogul	:	1.15 2013
Xtriage (Phenix)	:	dev-1439
EDS	:	stable21828
Percentile statistics	:	21802
Refmac	:	5.8.0049
CCP4	:	6.3.0 (Settle)
Ideal geometry (proteins)	:	Engh & Huber (2001)
Ideal geometry (DNA, RNA)	:	Parkinson et. al. (1996)
Validation Pipeline (wwPDB-VP)	:	stable21828

1 Overall quality at a glance (i)

The reported resolution of this entry is 1.85 Å.

Percentile scores (ranging between 0-100) for global validation metrics of the entry are shown in the following graphic. The table shows the number of entries on which the scores are based.

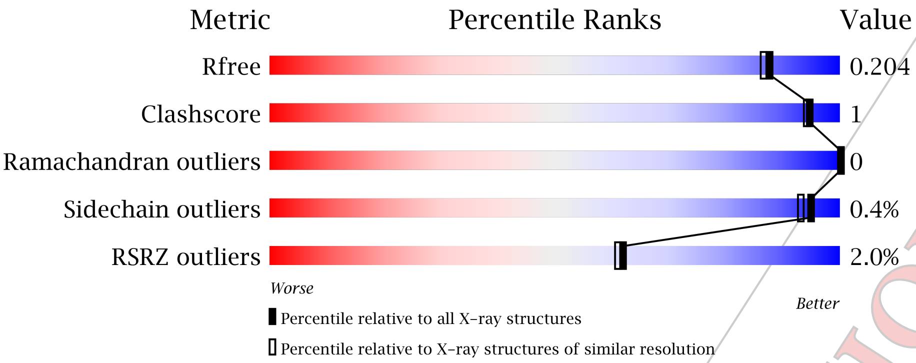

Metric	Whole archive (#Entries)	Similar resolution (#Entries, resolution range(Å))
R_{free}	66076	1269 (1.86-1.86)
Clashscore	79847	1470 (1.86-1.86)
Ramachandran outliers	78256	1451 (1.86-1.86)
Sidechain outliers	78232	1451 (1.86-1.86)
RSRZ outliers	66103	1269 (1.86-1.86)

The table below summarises the geometric issues observed across the polymeric chains and their fit to the electron density. The red, orange, yellow and green segments on the lower bar indicate the fraction of residues that contain outliers for >=3, 2, 1 and 0 types of geometric quality criteria. The upper red bar (where present) indicates the fraction of residues that have poor fit to the electron density.

Mol	Chain	Length	Quality of chain
1	F	375	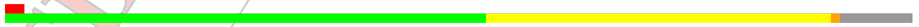

CONFIDENTIAL VALIDATION REPORT

2 Entry composition (i)

There are 4 unique types of molecules in this entry. The entry contains 3022 atoms, of which 0 are hydrogen and 0 are deuterium.

In the tables below, the ZeroOcc column contains the number of atoms modelled with zero occupancy, the AltConf column contains the number of residues with at least one atom in alternate conformation and the Trace column contains the number of residues modelled with at most 2 atoms.

- Molecule 1 is a protein called Farnesyl pyrophosphate synthase.

Mol	Chain	Residues	Atoms					ZeroOcc	AltConf	Trace
			Total	C	N	O	S			
1	F	344	2788	1802	455	519	12	0	5	0

There are 22 discrepancies between the modelled and reference sequences:

Chain	Residue	Modelled	Actual	Comment	Reference
F	-21	MET	-	EXPRESSION TAG	UNP P14324
F	-20	GLY	-	EXPRESSION TAG	UNP P14324
F	-19	SER	-	EXPRESSION TAG	UNP P14324
F	-18	SER	-	EXPRESSION TAG	UNP P14324
F	-17	HIS	-	EXPRESSION TAG	UNP P14324
F	-16	HIS	-	EXPRESSION TAG	UNP P14324
F	-15	HIS	-	EXPRESSION TAG	UNP P14324
F	-14	HIS	-	EXPRESSION TAG	UNP P14324
F	-13	HIS	-	EXPRESSION TAG	UNP P14324
F	-12	HIS	-	EXPRESSION TAG	UNP P14324
F	-11	SER	-	EXPRESSION TAG	UNP P14324
F	-10	SER	-	EXPRESSION TAG	UNP P14324
F	-9	GLY	-	EXPRESSION TAG	UNP P14324
F	-8	ARG	-	EXPRESSION TAG	UNP P14324
F	-7	GLU	-	EXPRESSION TAG	UNP P14324
F	-6	ASN	-	EXPRESSION TAG	UNP P14324
F	-5	LEU	-	EXPRESSION TAG	UNP P14324
F	-4	TYR	-	EXPRESSION TAG	UNP P14324
F	-3	PHE	-	EXPRESSION TAG	UNP P14324
F	-2	GLN	-	EXPRESSION TAG	UNP P14324
F	-1	GLY	-	EXPRESSION TAG	UNP P14324
F	0	HIS	-	EXPRESSION TAG	UNP P14324

- Molecule 2 is [({5-[4-(CYCLOPROPYLOXY)PHENYL]PYRIDIN-3-YL}AMINO)METHANEDIYL]BIS(PHOSPHONICACID) (three-letter code: JD5) (formula: $C_{15}H_{18}N_2O_7P_2$).

CONFIDENTIAL VALIDATION REPORT

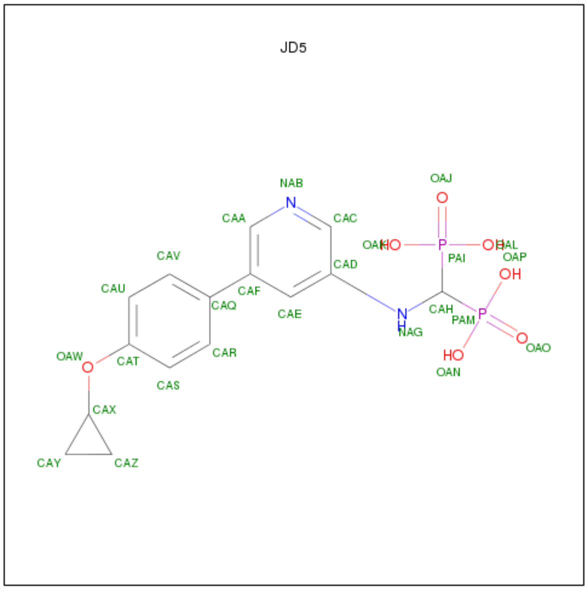

Mol	Chain	Residues	Atoms	ZeroOcc	AltConf
2	F	1	Total C N O P<br>26 15 2 7 2	0	0

- Molecule 3 is MAGNESIUM ION (three-letter code: MG) (formula: Mg).

Mol	Chain	Residues	Atoms	ZeroOcc	AltConf
3	F	3	Total Mg<br>3 3	0	0

- Molecule 4 is water.

Mol	Chain	Residues	Atoms	ZeroOcc	AltConf
4	F	205	Total O<br>205 205	0	0

CONFIDENTIAL VALIDATION REPORT

3 Residue-property plots (i)

These plots are drawn for all protein, RNA and DNA chains in the entry. The first graphic for a chain summarises the proportions of errors displayed in the second graphic. The second graphic shows the sequence view annotated by issues in geometry and electron density. Residues are color-coded according to the number of geometric quality criteria for which they contain at least one outlier: green = 0, yellow = 1, orange = 2 and red = 3 or more. A red dot above a residue indicates a poor fit to the electron density (RSRZ > 2). Stretches of 2 or more consecutive residues without any outlier are shown as a green connector. Residues present in the sample, but not in the model, are shown in grey.

- Molecule 1: Farnesyl pyrophosphate synthase

Chain F:

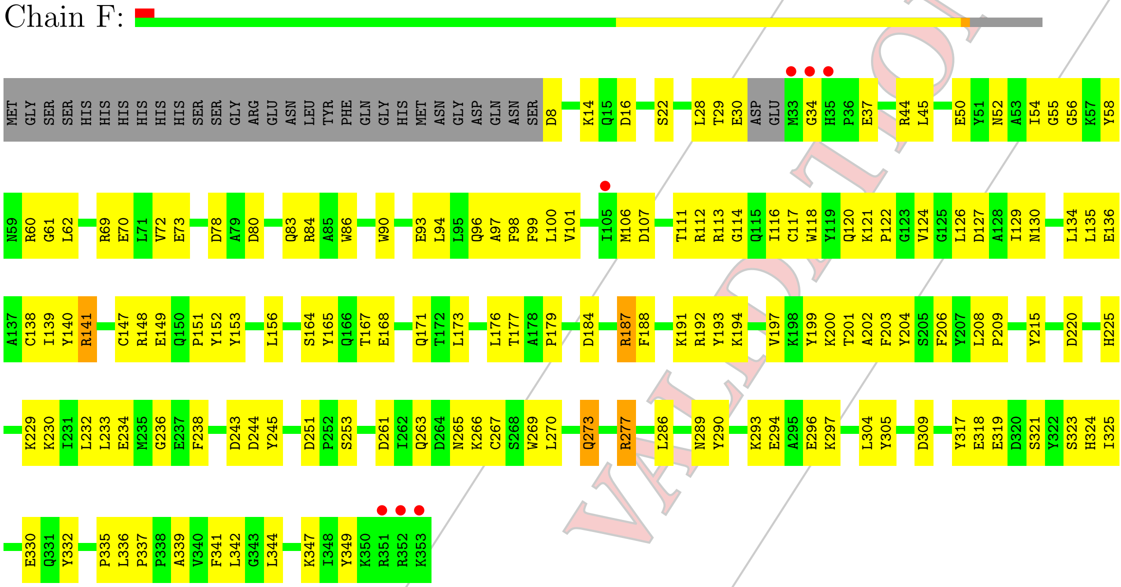

CONFIDENTIAL VALIDATION REPORT

4 Data and refinement statistics (i)

Property	Value	Source
Space group	P 41 21 2	Depositor
Cell constants<br>a, b, c, α, β, γ	110.51Å 110.51Å 66.97Å<br>90.00° 90.00° 90.00°	Depositor
Resolution (Å)	50.85 – 1.85<br>49.42 – 1.85	Depositor<br>EDS
% Data completeness<br>(in resolution range)	99.2 (50.85-1.85)<br>99.2 (49.42-1.85)	Depositor<br>EDS
R_{merge}	0.04	Depositor
R_{sym}	(Not available)	Depositor
$< I/\sigma(I) >$ [1]	4.65 (at 1.86Å)	Xtriage
Refinement program	REFMAC 5.8.0049	Depositor
R, R_{free}	0.165 , 0.196<br>0.173 , 0.204	Depositor<br>DCC
R_{free} test set	1781 reflections (5.27%)	DCC
Wilson B-factor (Å^2)	29.1	Xtriage
Anisotropy	0.245	Xtriage
Bulk solvent k_{sol}(e/Å^3), B_{sol}(Å^2)	0.37 , 45.7	EDS
Estimated twinning fraction	No twinning to report.	Xtriage
L-test for twinning	$< \|L\| > = 0.49$, $< L^2 > = 0.32$	Xtriage
Outliers	0 of 35583 reflections	Xtriage
F_o,F_c correlation	0.96	EDS
Total number of atoms	3022	wwPDB-VP
Average B, all atoms (Å^2)	37.0	wwPDB-VP

Xtriage's analysis on translational NCS is as follows: *The largest off-origin peak in the Patterson function is 4.08% of the height of the origin peak. No significant pseudotranslation is detected.*

[1] Intensities estimated from amplitudes.

CONFIDENTIAL VALIDATION REPORT

5 Model quality (i)

5.1 Standard geometry (i)

Bond lengths and bond angles in the following residue types are not validated in this section: JD5, MG

The Z score for a bond length (or angle) is the number of standard deviations the observed value is removed from the expected value. A bond length (or angle) with $|Z| > 2$ is considered an outlier worth inspection. RMSZ is the root-mean-square of all Z scores of the bond lengths (or angles).

Mol	Chain	Bond lengths		Bond angles	
		RMSZ	#$\|Z\|$ >2	RMSZ	#$\|Z\|$ >2
1	F	0.95	97/2429 (4.0%)	0.97	124/2729 (4.5%)

The worst 5 of 97 bond length outliers are listed below:

Mol	Chain	Res	Type	Atoms	Z	Observed(Å)	Ideal(Å)
1	F	136	GLU	CD-OE2	6.43	1.32	1.25
1	F	55	GLY	N-CA	6.30	1.55	1.46
1	F	22	SER	CB-OG	-4.14	1.36	1.42
1	F	168	GLU	CD-OE1	-4.00	1.21	1.25
1	F	330	GLU	CD-OE2	3.97	1.30	1.25

The worst 5 of 124 bond angle outliers are listed below:

Mol	Chain	Res	Type	Atoms	Z	Observed(^{o})	Ideal(^{o})
1	F	192	ARG	NE-CZ-NH1	-6.53	117.03	120.30
1	F	141	ARG	NE-CZ-NH1	-5.09	117.76	120.30
1	F	80	ASP	CB-CG-OD1	4.80	122.62	118.30
1	F	8	ASP	CB-CG-OD1	4.59	122.43	118.30
1	F	141	ARG	CG-CD-NE	-4.45	102.45	111.80

There are no chirality outliers.

There are no planarity outliers.

5.2 Close contacts (i)

In the following table, the Non-H and H(model) columns list the number of non-hydrogen atoms and hydrogen atoms in the chain respectively. The H(added) column lists the number of hydrogens added by MolProbity. The Clashes column lists the number of clashes within the asymmetric unit, and the number in parentheses is this value normalized per 1000 atoms of the molecule in the

CONFIDENTIAL VALIDATION REPORT

chain. The Symm-Clashes column gives symmetry related clashes, in the same way as for the Clashes column.

Mol	Chain	Non-H	H(model)	H(added)	Clashes	Symm-Clashes
1	F	2788	0	0	4	0
2	F	26	0	0	0	0
3	F	3	0	0	0	0
4	F	205	0	0	3	0
All	All	3022	0	0	4	0

Clashscore is defined as the number of clashes calculated for the entry per 1000 atoms (including hydrogens) of the entry. The overall clashscore for this entry is 1.

All close contacts within the same asymmetric unit are listed below.

Atom-1	Atom-2	Distance(Å)	Clash(Å)
1:F:324:HIS:ND1	4:F:701:HOH:O	2.33	0.54
1:F:187:ARG:NH1	4:F:696:HOH:O	2.44	0.49
1:F:141:ARG:CD	4:F:661:HOH:O	2.64	0.45
1:F:273:GLN:OE1	1:F:277:ARG:NH1	2.52	0.42

There are no symmetry-related clashes.

5.3 Torsion angles

5.3.1 Protein backbone (i)

In the following table, the Percentiles column shows the percent Ramachandran outliers of the chain as a percentile score with respect to all X-ray entries followed by that with respect to entries of similar resolution. The Analysed column shows the number of residues for which the backbone conformation was analysed, and the total number of residues.

Mol	Chain	Analysed	Favoured	Allowed	Outliers	Percentiles	
1	F	345/375 (92%)	341 (99%)	4 (1%)	0	100	100

There are no Ramachandran outliers to report.

5.3.2 Protein sidechains (i)

In the following table, the Percentiles column shows the percent sidechain outliers of the chain as a percentile score with respect to all X-ray entries followed by that with respect to entries of similar resolution. The Analysed column shows the number of residues for which the sidechain conformation was analysed, and the total number of residues.

CONFIDENTIAL VALIDATION REPORT

Mol	Chain	Analysed	Rotameric	Outliers	Percentiles	
1	F	289/322 (90%)	288 (100%)	1 (0%)	96	94

All residues with a non-rotameric sidechain are listed below:

Mol	Chain	Res	Type
1	F	204	TYR

Some sidechains can be flipped to improve hydrogen bonding and reduce clashes. There are no such sidechains identified.

5.3.3 RNA (i)

There are no RNA chains in this entry.

5.4 Non-standard residues in protein, DNA, RNA chains (i)

There are no non-standard protein/DNA/RNA residues in this entry.

5.5 Carbohydrates (i)

There are no carbohydrates in this entry.

5.6 Ligand geometry (i)

Of 4 ligands modelled in this entry, 3 are monoatomic - leaving 1 for Mogul analysis.

In the following table, the Counts columns list the number of bonds (or angles) for which Mogul statistics could be retrieved, the number of bonds (or angles) that are observed in the model and the number of bonds (or angles) that are defined in the chemical component dictionary. The Link column lists molecule types, if any, to which the group is linked. The Z score for a bond length (or angle) is the number of standard deviations the observed value is removed from the expected value. A bond length (or angle) with $|Z| > 2$ is considered an outlier worth inspection. RMSZ is the root-mean-square of all Z scores of the bond lengths (or angles).

Mol	Type	Chain	Res	Link	Bond lengths			Bond angles		
					Counts	RMSZ	$\#\|Z\| > 2$	Counts	RMSZ	$\#\|Z\| > 2$
2	JD5	F	401	3	28,28,28	2.21	11 (39%)	42,42,42	2.52	19 (45%)

In the following table, the Chirals column lists the number of chiral outliers, the number of chiral centers analysed, the number of these observed in the model and the number defined in the chemical component dictionary. Similar counts are reported in the Torsion and Rings columns. '-' means

CONFIDENTIAL VALIDATION REPORT

no outliers of that kind were identified.

Mol	Type	Chain	Res	Link	Chirals	Torsions	Rings
2	JD5	F	401	3	-	0/24/26/26	0/2/3/3

The worst 5 of 11 bond length outliers are listed below:

Mol	Chain	Res	Type	Atoms	Z	Observed(Å)	Ideal(Å)
2	F	401	JD5	PAM-CAH	6.03	1.88	1.83
2	F	401	JD5	PAI-CAH	4.90	1.78	1.83
2	F	401	JD5	CAZ-CAX	3.85	1.36	1.48
2	F	401	JD5	PAI-OAJ	3.55	1.56	1.49
2	F	401	JD5	CAZ-CAY	2.48	1.39	1.48

The worst 5 of 19 bond angle outliers are listed below:

Mol	Chain	Res	Type	Atoms	Z	Observed(^{o})	Ideal(^{o})
2	F	401	JD5	OAW-CAX-CAZ	10.12	93.53	115.82
2	F	401	JD5	PAM-CAH-PAI	4.66	107.99	116.14
2	F	401	JD5	OAK-PAI-OAJ	3.79	103.88	113.60
2	F	401	JD5	CAR-CAQ-CAF	3.53	127.61	121.41
2	F	401	JD5	OAK-PAI-CAH	3.45	113.31	104.77

There are no chirality outliers.

There are no torsion outliers.

There are no ring outliers.

5.7 Other polymers (i)

There are no such residues in this entry.

CONFIDENTIAL VALIDATION REPORT

6 Fit of model and data (i)

6.1 Protein, DNA and RNA chains (i)

In the following table, the column labelled '#RSRZ> 2' contains the number (and percentage) of RSRZ outliers, followed by percent RSRZ outliers for the chain as percentile scores relative to all X-ray entries and entries of similar resolution. The OWAB column contains the minimum, median, 95th percentile and maximum values of the occupancy-weighted average B-factor per residue. The column labelled 'Q< 0.9' lists the number of (and percentage) of residues with an average occupancy less than 0.9.

Mol	Chain	Analysed	<RSRZ>	#RSRZ>2			OWAB(Å^2)	Q<0.9
1	F	344/375 (91%)	0.02	7 (2%)	62	61	21, 34, 62, 80	0

The worst 5 of 7 RSRZ outliers are listed below:

Mol	Chain	Res	Type	RSRZ
1	F	34	GLY	4.6
1	F	33	MET	4.2
1	F	352	ARG	3.6
1	F	351	ARG	2.7
1	F	353	LYS	2.3

6.2 Non-standard residues in protein, DNA, RNA chains (i)

There are no non-standard protein/DNA/RNA residues in this entry.

6.3 Carbohydrates (i)

There are no carbohydrates in this entry.

6.4 Ligands (i)

In the following table, the Atoms column lists the number of modelled atoms in the group and the number defined in the chemical component dictionary. LLDF column lists the quality of electron density of the group with respect to its neighbouring residues in protein, DNA or RNA chains. The B-factors column lists the minimum, median, 95th percentile and maximum values of B factors of atoms in the group. The column labelled 'Q< 0.9' lists the number of atoms with occupancy less than 0.9.

Mol	Type	Chain	Res	Atoms	RSR	LLDF	B-factors(Å^2)	Q<0.9
3	MG	F	403	1/1	0.09	0.05	23,23,23,23	0

Continued on next page...

CONFIDENTIAL VALIDATION REPORT

Continued from previous page...

Mol	Type	Chain	Res	Atoms	RSR	LLDF	B-factors(Å^2)	Q<0.9
2	JD5	F	401	26/26	0.12	0.00	20,24,38,39	0
3	MG	F	402	1/1	0.10	-1.03	22,22,22,22	0
3	MG	F	404	1/1	0.09	-6.61	24,24,24,24	0

6.5 Other polymers (i)

There are no such residues in this entry.

CONFIDENTIAL VALIDATION REPORT
