# Supplementary figures and images for: Crystallographic and thermodynamic characterization of phenylaminopyridine bisphosphonates binding to human farnesyl pyrophosphate synthase
Source: PLoS One. 2017 Oct 16;12(10):e0186447. doi: 10.1371/journal.pone.0186447 (PMC5643135; doi:10.1371/journal.pone.0186447)

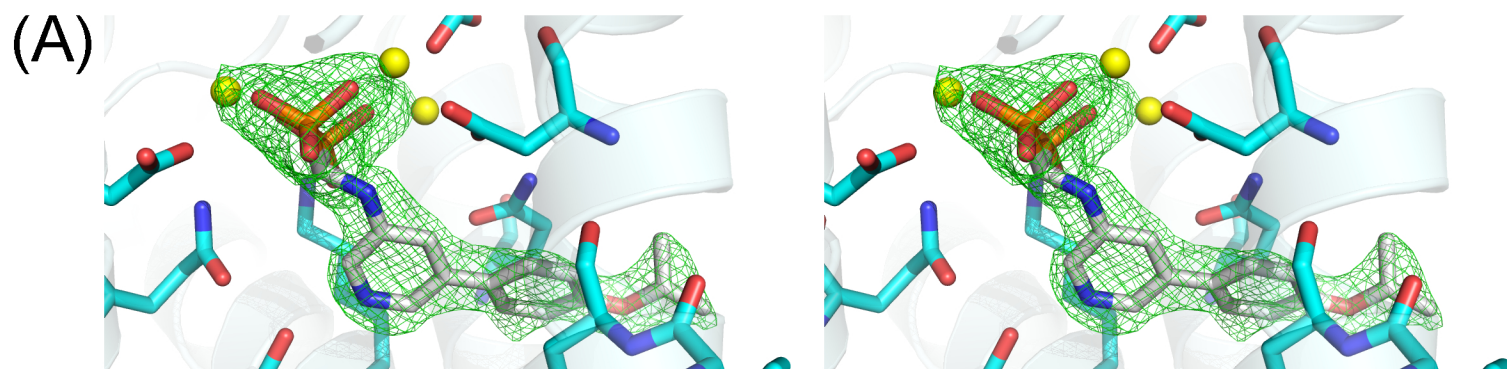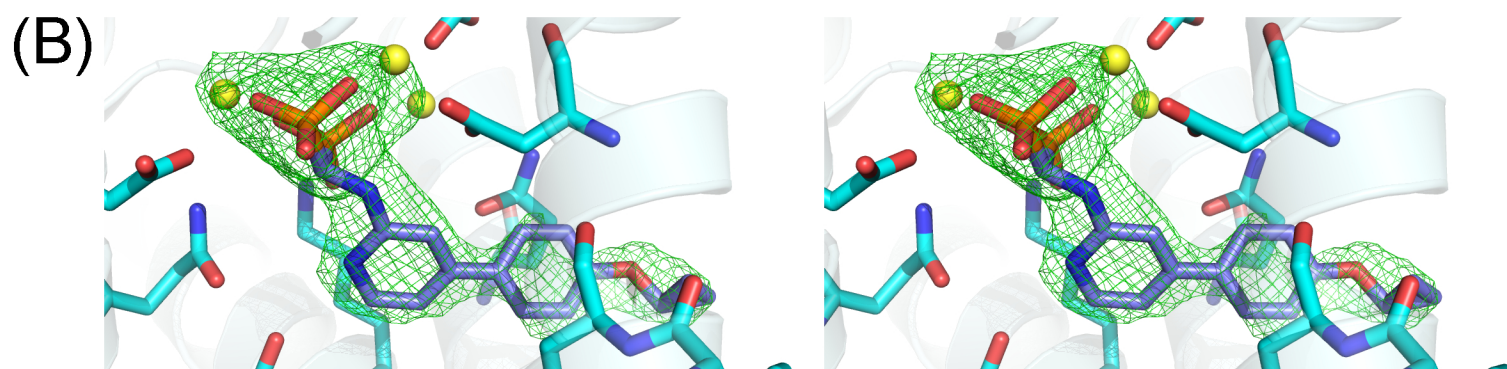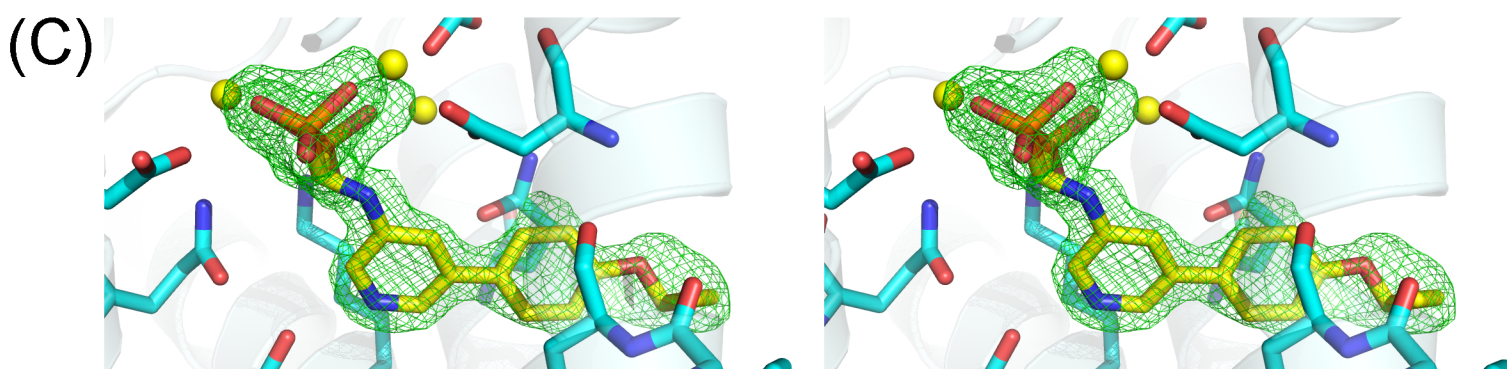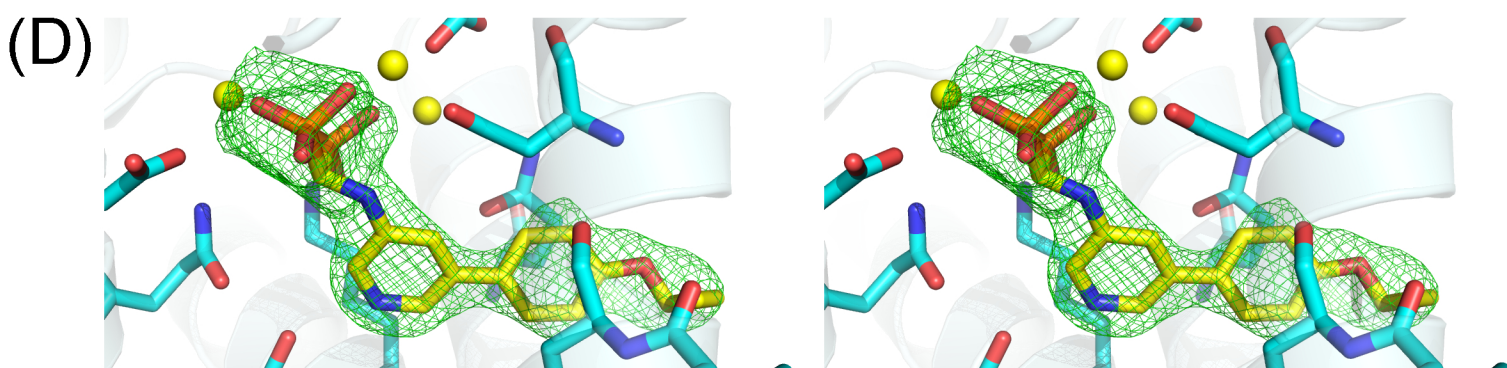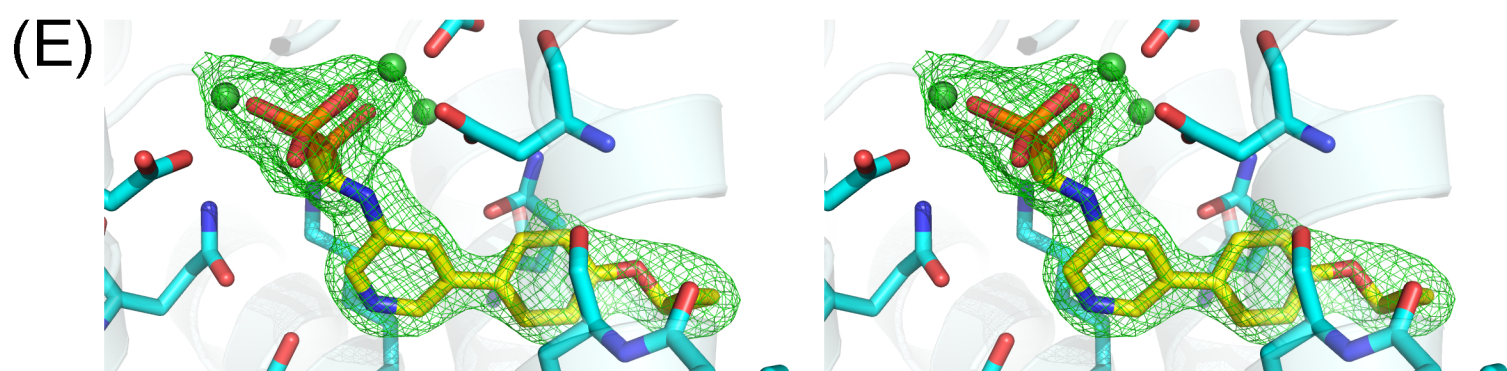

Supplement: S1 Fig — (A) JDS05119 (PDB entry 4PVY); (B) YS05035 (PDB entry 4PVX); (C), (D), and (E) JDS05120 (PDB entries 4NFI, 4NFJ, and 4NFK, respectively). The green meshes represent the Fo-Fc electron density maps (3σ) generated by Fourier synthesis before ever modeling the ligands. Green spheres are Ni2+ ions. (PDF) [file pone.0186447.s006.pdf]
